# 3-Anhydro-6-hydroxy-ophiobolin A displays high *in vitro* and *in vivo* efficacy against influenza A virus infection

**DOI:** 10.1007/s13238-016-0325-y

**Published:** 2016-10-14

**Authors:** Song Wang, Xiaoqin Luo, Ruoxiang Yan, Quanxin Wang, Qiuyue Qi, Xiaojuan Chi, Lanlan Zhang, Ziding Yu, Binxiang Cai, Ji-Long Chen, Hongwei Liu

**Affiliations:** 1 College of Animal Science, Fujian Agriculture and Forestry University, Fuzhou, 350002 China; 2 Institute of Microbiology, Chinese Academy of Sciences, Beijing, 100101 China

**Dear Editor,**

Influenza A virus (IAV) is an enveloped negative-strand RNA virus containing eight RNA segments that belongs to the family Orthomyxoviridae and can cause acute respiratory infection in humans and animals. Although vaccination is one of the major means for prophylaxis of influenza virus infection, a particular influenza vaccine only confers protection for no more than a few years. In addition, the outbreak of novel influenza infection cannot be predicted (Gao et al., 2013). Thus, vaccine is unable to provide immediate protection against sudden influenza outbreaks with unknown identity. Development of antiviral drugs for therapeutic treatment is an important strategy to reduce the duration and severity of influenza. However, more and more drug-resistant influenza virus strains are emerging due to its antigenic drift or antigenic shift, which increases the need for new antivirals.

Over the past decades, progresses have been made in developing small molecule compounds for treatment of influenza viral infection. For example, previous experiments demonstrated that the novel NF-kappaB inhibitor SC75741 significantly protected mice against infection with highly pathogenic avian influenza A viruses (HPAIV) of the H5N1 and H7N7 subtypes (Haasbach et al., 2013). The MEK inhibitor U0126, targeting the intracellular Raf/MEK/ERK signaling pathway, is able to suppress propagation of both the 2009 pandemic IAV and HPAIV *in vitro* and *in viv*o (Droebner et al., 2011). Moreover, two identified novel anti-IAV agents, obatoclax and gemcitabine possess broad-spectrum antiviral activity. Obatoclax can inhibit IAV uptake and gemcitabine can suppress viral RNA transcription and replication (Denisova et al., 2012; Planz, 2013). Influenza virus utilizes cellular machinery for the replication and assembly of viral components and the release of progeny virions. Recent genome-wide RNAi screening has identified several host genes and molecular networks crucial for the viral replication (Karlas et al., 2010; Watanabe et al., 2010), providing potential ways for anti-influenza therapy.

In our ongoing search for new bioactive compounds from fungi, we have recently reported 3-anhydro-6-hydroxy-ophiobolin A (L435-3), a new sesterterpene with antibacterial activities from the phytopathogenic fungus *Bipolaris oryzae* (Wang et al., 2013). In this study, we further examined the potential anti-influenza activity of L435-3 *in vitro* and *in vivo*. First, A549 cells were infected with influenza virus strain WSN virus and then treated with or without L435-3. We found that treatment with L435-3 could inhibit the IAV replication at very low concentration (Fig. 1A), indicating that L435-3 may be a potential therapeutic agent for treatment of influenza virus infections.

**Figure 1:**
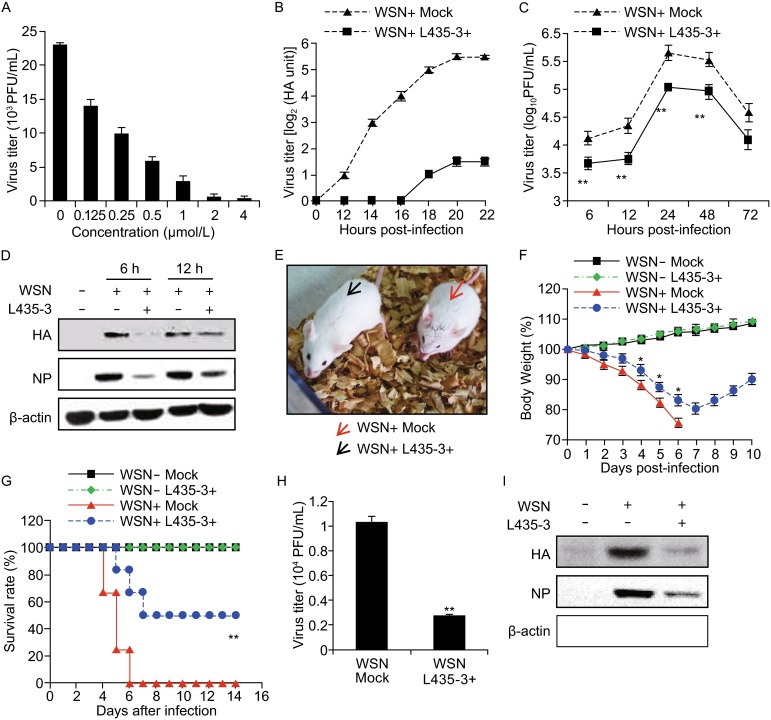
**L435-3 inhibits IAV replication** ***in vitro*** **and** ***in vivo***. (A) A549 Cells were infected with WSN viruses and further treated with different concentrations of L435-3 for 12 h. Progeny virus titer was determined by plaque assay. (B and C) A549 cells infected with WSN viruses were mock treated or treated with L435-3 (0.5 μmol/L) for indicated times. The supernatants of cell culture were examined for the viral titers by hemagglutinin assay (B) and plaque assay (C). (D) A549 cells infected with WSN virus were mock treated or treated with L435-3 (0.5 μmol/L), then the cells were harvested at the indicated times, and followed by analysis of Western blotting with indicated antibodies. (E) BALB/c mice infected with WSN viruses (5 × 10^4^ PFU/mouse) were mock treated or inoculated intranasally with L435-3 (0.3 mg/kg). Shown is a representative photograph of the two differently treated mice. (F) Shown are body weight changes of control mice, WSN and/or L435-3 treated mice. *P < 0.05, difference between WSN+ L435-3− and WSN+ L435-3+ groups by daily examination. (G) Survival rate of control mice, WSN and/or L435-3 treated mice. The mice were monitored for up to 14 d. Survival curves were compared using a log-rank test (GraphPad Prism 5). **P < 0.01, difference between WSN+ L435-3- and WSN+ L435-3+ groups. (H) WSN-infected mice were mock treated or treated with L435-3 (0.3 mg/kg) for 3 days. Then the mice were sacrificed and viral titers in the lungs were measured by plaque assay. (I) WSN-infected mice were treated with L435-3 as described in (H). Then the lungs were homogenized, followed by analysis of Western blotting with indicated antibodies. * *P* < 0.05, **P < 0.01

To further confirm the inhibitory effect of L435-3 on IAV infection, we performed several experiments using A549 and MDCK cells infected with WSN or PR8 virus. As shown in Table S1, L435-3 displayed high activity against WSN or PR8 virus infection in MDCK cells, although its activity is lower than that of zanamivir. Then A549 cells were infected with WSN at a multiplicity of infection (MOI) of 0.2, and treated with 0.5 μM L435-3 at 1 h post-infection. Indeed, 0.5 μM L435-3 showed little cytotoxicity to A549 cells, and the influenza virus titers were markedly reduced by L435-3 treatment (Fig. 1B and 1C). Using Western blotting, we further confirmed that L435-3 treatment significantly inhibited influenza virus replication, since the levels of both viral HA and NP were markedly reduced in WSN-infected A549 cells treated with L435-3 (Fig. 1D). Together, these experiments demonstrate that L435-3 is a potent inhibitor of IAV replication in host cell.

Next, we investigated the anti-influenza virus activity of L435-3 *in vivo*. To this end, Balb/c mice were separated into four groups (12 mice/each group). Two groups of mice were infected with WSN and then inoculated intranasally with L435-3 (0.3 mg/kg/mouse) or mock control. The other two groups of mice were only treated with L435-3 or mock control. All mice were monitored for body weight change and survival rate. As expected, WSN challenge resulted in obvious flu symptoms in all mice on day 2 post-infection. However, the symptoms were remarkably less severe in L435-3 treated group (Fig. 1E). The mice without L435-3 treatment showed a more weight loss as compared to L435-3 treated group during the IAV infection (Fig. 1F). To further determine the efficacy of L435-3, we compared the mortality between these groups over a 14-day infection period. The results displayed that the control group mice began to die on day 4 post-infection and all mice died within 6 days after infection. However, only 50% mice died in L435-3 treated group over the whole time course. Of note, no mice died in uninfected group (Fig. 1G).

To determine whether L435-3 affected pathological changes induced by WSN infection, the mice were sacrificed and dissected on day 4 post-infection. We observed that the pulmonary lesions in the L435-3 treated group were less than that in the control group without L435-3 treatment. Additionally, the atrophy of spleens and thymuses in L435-3 treated group was less severe than that in the control group (Fig. S1A). Pathohistological analysis revealed that the lung tissues in L435-3 treated group displayed less inflammation, interstitial edema, and lung consolidation than the control group (Fig. S1B). The spleen tissues in L435-3 treated group showed that the areas of white pulp were increased, and there were clearer boundaries between white pulps and red pulps than the control group (Fig. S2C). Together, these observations suggest that L435-3 has a profound inhibitory effect on WSN infection of mice.

Because L435-3 showed considerable protection against IAV infection in mice, we further determined whether L435-3 inhibited the viral replication in mice. Consistent with our *in vitro* data presented above, treatment with L435-3 significantly reduced the viral titers in the lungs of WSN-infected mice (Fig. 1H), and the protein levels of HA and NP were markedly lower in the L435-3 treated group than those in the control group (Fig. 1I). Collectively, these results reveal that L435-3 significantly impairs the viral replication *in vivo* during the IAV infection of mice.

In an attempt to explore the mechanisms by which L435-3 inhibits influenza virus replication, cDNA microarray analysis was performed to determine the differentially expressed genes in IAV-infected A549 cells in response to L435-3 treatment (http://www.ncbi.nlm.nih.gov/geo/; GenBank accession number GSE58741). Treatment with L435-3 resulted in up-regulation of 1027 genes, and down-regulation of 1047 genes in IAV-infected A549 cells. Interestingly, we found that many genes were involved in innate immunity and inflammatory response.

To verify the cDNA microarray data, RT-PCR and quantitative real-time PCR were employed. We observed that the expressions of *IL28* and *IL29* were markedly up-regulated by L435-3 treatment at 6 or 12 h after WSN infection (Fig. 2A–C). Moreover, the expression levels of several interferon-stimulated genes (ISGs) were measured by quantitative real-time PCR. As shown in Fig. S2, the expressions of *ISG15, ISG20* and *OASL* were significantly increased in WSN-infected A549 cells treated with L435-3 as compared to the control.

**Figure 2:**
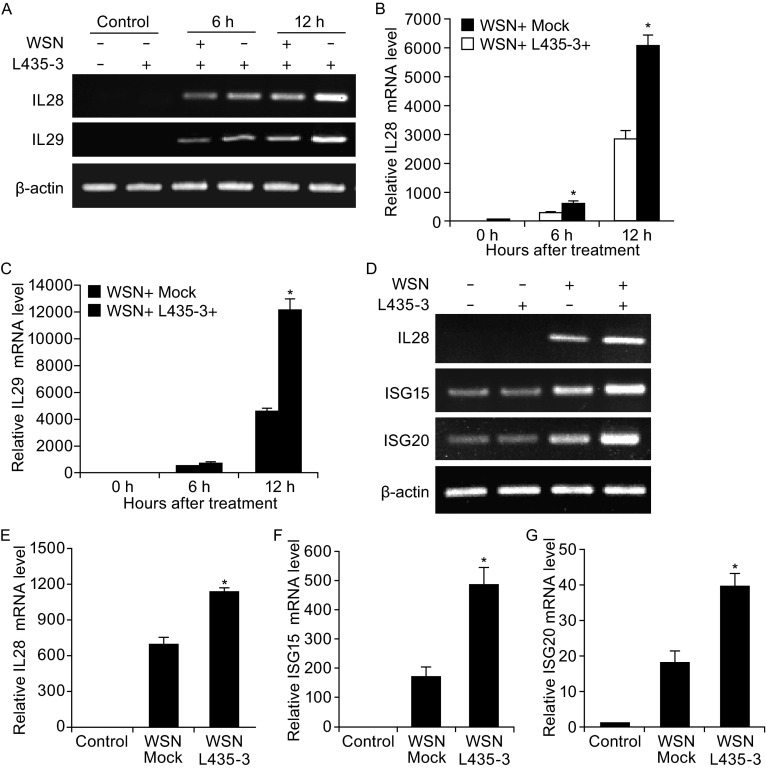
**L435-3 treatment increases the expression of type III interferons and ISGs both in A549 cells and in mice infected with IAV**. (A) WSN-infected A549 cells were treated with or without L435-3 (0.5 μmol/L) for 6 h and 12 h, and then the mRNA levels of indicated genes were analyzed by RT-PCR. (B and C) WSN-infected A549 cells were treated with or without L435-3 as described in (A). The mRNA levels of *IL28* (B) and *IL29* (C) were analyzed by quantitative real-time PCR. (D) WSN-infected BALB/c mice were treated with or without L435-3 (0.3 mg/kg/mouse) for 3 days. Then the mice were sacrificed, and the mRNA levels of indicated genes in mouse lung were detected by RT-PCR. (E, F and G) WSN-infected BALB/c mice were treated as described in (D). The mRNA levels of *IL28* (E), *ISG15* (F) and *ISG20* (G) in mouse lungs were detected by quantitative real-time PCR. *P < 0.05

Next, we tested whether L435-3-mediated inhibition of IAV replication in mice was caused by increased expression of interferons. Consistent with *in vitro* observations presented above, L435-3 treatment led to an increase in expression levels of *IL28*, *ISG15* and *ISG20* in WSN infected mice by both RT-PCR (Fig. 2D) and quantitative real-time PCR (Fig. 2E–G). These results suggest that L435-3 inhibits IAV replication likely through increasing the production of type III interferons and some ISGs.

Influenza virus is still a threat to public health and world economy. Although two classes of antiviral agents targeting M2 or NA are used in the clinical treatment of influenza viral infection, an increasing number of drug-resistant viruses have emerged (Regoes and Bonhoeffer, 2006; Cheng et al., 2009; Hurt et al., 2009; Moscona, 2009). Therefore, the development of novel anti-IAV drug has become an urgent task to combat against influenza viruses. In this study, we identified L435-3, a new derivative of ophiobolins A from fungus B. oryzae, as a potent inhibitor that strongly suppresses IAV infection.

Ophiobolins are a group of phytotoxic sesterterpenoids and secondary metabolites produced by the phytopathogenic fungi that attack maize, rice, and sorghum. They possesses a broad spectrum of inhibitory activity against fungi, bacteria, and nematodes, and cytotoxic activity against cancer cells (Au et al., 2000; Phuwapraisirisan et al., 2007; Yang et al., 2012; Wang et al., 2013). As a derivative of ophiobolins, L435-3 has a good antimicrobial activity against Bacille Calmette-Guerin, Bacillus subtilis, Staphylococcus aureus, and methicillin-resistant Staphylococcus aureus. Moreover, it exhibits potent antiproliferative activity against K562 and HepG2 cell lines (Wang et al., 2013). In this study, our results represent the first report of ophiobolin derivative L435-3 that displays high *in vitro* and *in vivo* efficacy against IAV infection. However, whether this compound possesses activity against other viruses remains to be further determined.

Our study has also addressed the mechanism by which L435-3 may affect pathogenesis of influenza infections. The microarray analysis showed that L435-3 treatment could induce higher level expressions of type III IFNs and several ISGs both in cell line and mice during the IAV infection, and thereby established an antiviral state in host. This finding was further confirmed by RT-PCR and real-time PCR.

In summary, our results identified L435-3 as a novel and potent inhibitor against IAV infection through enhancing the host immunity *in vitro* and *in vivo*. However, there might exist other mechanisms by which L435-3 defends against IAV infection, and the precise mechanisms underlying function of L435-3 remain to be better understood. As a promoter of host immune responses to IAV infection, L435-3 can likely inhibit the infection of influenza viruses that are resistant to oseltamivir and/or amantadine. Therefore, L435-3 may be a valuable candidate for development of a novel anti-influenza agent.

## Electronic supplementary material

Below is the link to the electronic supplementary material.

Supplementary material 1 (PDF 407 kb)
