# Supplementary material for: 3-Anhydro-6-hydroxy-ophiobolin A displays high in vitro and in vivo efficacy against influenza A virus infection
Source: Protein Cell. 2016 Oct 14;7(11):839–43. doi: 10.1007/s13238-016-0325-y (PMC5084159; doi:10.1007/s13238-016-0325-y)
Supplement: Supplementary file 1 — Supplementary material 1 (PDF 407 kb) [file 13238_2016_325_MOESM1_ESM.pdf]

SUPPLEMENTAL INFORMATION

MATERIALS AND METHODS

Ethics statement

The mouse experimental design and protocols used in this study were approved by “the regulation of College of Animal Sciences, Fujian Agriculture and Forestry University of Research Ethics Committee” (Permit Number PZCASFAFU2014001). All mouse experimental procedures were performed in accordance with the Regulations for the Administration of Affairs Concerning Experimental Animals approved by the State Council of People’s Republic of China.

Reagents and antibodies

3-(4,5)-dimethylthiahiazo (-z-y1)-3,5-di-phenytetrazoliumromide (MTT) was purchased from Sigma. The small molecule compounds including L435-3 were obtained as previously described (Wang et al., 2013). The purity of these compounds is over 95% on the basis of HPLC analysis. The structure of L435-3 was confirmed by ^{1}H and ^{13}C NMR spectra. All other antibodies were obtained as previously described (Wang et al., 2014; Wei et al., 2014).

Influenza virus and infection

Mouse-adapted influenza virus strain A/WSN/33 (H1N1) (WSN) was prepared and the viral infection was performed as previously described (Wang et al., 2014; Wei et al., 2014). The MLD50 of WSN virus was approximately 5×10^4 PFU under our conditions. A549 human alveolar epithelial cells and

Madin-Darby canine kidney (MDCK) cells were used in this study. These cells were maintained in Dulbecco’s Modified Eagle’s Medium (DMEM) containing 10% fetal bovine serum (FBS) supplemented with penicillin (100 U/ml) and streptomycin (100 μg/ml). For infection, cells were washed with phosphate-buffered saline (PBS) and infected with the virus using indicated multiplicity of infection (MOI).

Mouse experiments

For virus infection, female BALB/c mice (5 to 6 weeks old, 20 to 22 g) were inoculated intranasally with 5×10^4 PFU of WSN virus. For L435-3 treatment, at 1 h post-infection (p.i.), mice were inoculated intranasally with L435-3 using 0.3 mg/kg body weight. At the indicated time, mice were euthanized and the lungs were homogenized with cold PBS, and centrifuged at 3,000 rpm for 10 minutes at 4 °C. Ten-fold dilutions of lung homogenates were prepared in PBS and used for plaque assay.

Measurement of pharmacological parameters in vitro

The toxic concentration 50% (TC_{50}) of L435-3 or zanamivir was determined using MDCK cells. 5×10^4 cells were seeded in a 96-well plate and incubated with different concentration of L435-3 or zanamivir for 24 h. After incubation, toxic concentration was measured by MTT assay according to the manufactures' instructions (Sigma). To determine the inhibitory concentration 50% (IC_{50}) of L435-3 or zanamivir against influenza A virus, 5×10^4 MDCK cells per well in a 96-well plate were infected with WSN virus. After 1 hour, virus

inoculum was discarded and the infected cells were treated with L435-3 or zanamivir in different concentrations for 24 h. Virus titers in the supernatants of treated cells were measured by plaque assay. All experiments were performed in triplicates. Results were evaluated by GraphPad prism software.

RT-PCR, quantitative real-time PCR and Western blotting

Total RNA was extracted from cells or mice tissues using TRIzol reagent (Invitrogen, Carlsbad, CA). The isolated RNA was used to synthesize cDNA with M-MLV reverse transcriptase (Promega, Madison, WI), and followed by PCR using rTaq DNA Polymerase and quantitative PCR using SYBR Premix *Ex* Taq II (Takara, Tokyo, Japan) with the following primers: human *IL28* forward, 5’-AGCTGCAGGCCTTTAAGAGG-3’, and reverse, 5’-TCCAGAACCTTCAGCGTCAG-3’; mouse *IL28* forward, 5’-AGCTGCAGGCCTTCAAAAAG-3’, and reverse, 5’-TGGGAGTGAATGTGGCTCAG-3’; human *IL29* forward, 5’-CCAAGCCCACCACAACTGGG-3’, and reverse, 5’-TGTGGTGCAGGGTGTGAAGG-3’; human *ISG15* forward, 5’-GACCTGACGGTGAAGATGCTG-3’, and reverse, 5’-TGCTGCGGCCCTTGTTAT-3’; mouse *ISG15* forward, 5’-GGGGTAACGATTTCCTG-3’, and reverse, 5’- CATAGATGTTGCTGTGGC-3’; human *ISG20* forward, 5’-GTCCACGGTGCTGTGCTGTA-3’, and reverse, 5’-TCGTTGCCCTCGCATCTTC-3’; mouse *ISG20* forward, 5’-GAGATCACGGACTACAGAACC-3’, and reverse, 5’-

AGCCTCAGGGACACTCG-3'; human *OASL* forward, 5'-CCCTGAGGTCTATGTGAGC-3', and reverse, 5'-GTGAAGCCTTCGTCCAAC-3'. Other primers were described as previously reported (Wang et al., 2014). For Western blotting, samples were prepared and immunoblotting was performed as previously described (Wang et al., 2014). Briefly, samples were separated on SDS-polyacrylamide gel, transferred onto a nitrocellulose membrane, probed with antibodies as indicated.

Hemagglutinin assay and plaque assay

A549 cells were infected with WSN virus at the indicated multiplicity of infection. After adsorption for 1 h at 37 °C, the cells were washed with PBS and maintained in DMEM containing 2 μg/ml trypsin, and the supernatants of cell cultures were then harvested at the indicated times. For hemagglutinin assay, the supernatants were diluted with PBS and mixed with an equal volume of 0.5% chicken erythrocytes. The viral titers were counted from the highest dilution factors that produced a positive reading. For plaque assay, MDCK cells were infected with serial dilutions of the viruses. Cells were overlaid with DMEM containing 1.5% low melting point agarose and 2 μg/ml l-1-tosylamido-2-phenylethyl chloromethyl ketone (TPCK)-treated trypsin. After 72 h of incubation at 37 °C, plaques were stained with neutral red and counted. (Wang et al., 2012).

Histopathological analysis

Histopathological analysis was performed as described previously (Wang et al., 2014). Briefly, mouse tissues were fixed in 4% paraformaldehyde and embedded in paraffin. Then 4-mm-thick sections were prepared and stained with hematoxylin and eosin (H&E). Sections were examined under an Olympus BH-2 microscope (Tokyo, Japan).

Statistical analysis

Data represent the mean values ± standard errors (SE). Survival curves were analyzed using the log-rank test (GraphPad Prism 5). Other statistical analysis was performed by Student's *t* test. Differences were considered statistically significant with $P < 0.05$.

References

Wang QX, Yang JL, Qi QY, Bao L, Yang XL, Liu MM, Huang P, Zhang LX, Chen JL, Cai L, et al. (2013) 3-Anhydro-6-hydroxy-ophiobolin A, a new sesterterpene inhibiting the growth of methicillin-resistant Staphylococcus aureus and inducing the cell death by apoptosis on K562, from the phytopathogenic fungus Bipolaris oryzae. Bioorg Med Chem Lett 23: 3547-3550

Wang S, Chi X, Wei H, Chen Y, Chen Z, Huang S, Chen JL (2014) Influenza A Virus-Induced Degradation of Eukaryotic Translation Initiation Factor 4B Contributes to Viral Replication by Suppressing IFITM3 Protein Expression. J Virol 88: 8375-8385

Wang S, Li H, Chen Y, Wei H, Gao GF, Liu H, Huang S, Chen JL (2012) Transport

of influenza virus neuraminidase (NA) to host cell surface is regulated by ARHGAP21 and Cdc42 proteins. J Biol Chem 287: 9804-9816

Wei H, Wang S, Chen Q, Chen Y, Chi X, Zhang L, Huang S, Gao GF, Chen JL (2014) Suppression of interferon lambda signaling by SOCS-1 results in their excessive production during influenza virus infection. PLoS pathog 10: e1003845

Supplementary Table 1. Inhibitory effects of L435-3 and Zanamivir on viral infection with WSN or PR8

Inhibitor	TC_{50} (μM)	WSN		PR8	
		IC_{50} (μM)	SI[a]	IC_{50} (μM)	SI[a]
**L435-3**	**5.219**	**0.365**	**14.299**	**0.391**	**13.348**
**Zanamivir**	**>100**	**3.176×10^{-3}**	**$>3.15\times10^{4}$**	**8.486×10^{-3}**	**$>1.18\times10^{4}$**

As described in Materials and methods, the toxic concentration 50% (TC_{50}) of L435-3 or zanamivir was determined in MDCK cells and measured by MTT assay according to the manufactures' instructions (Sigma). The inhibitory concentration 50% (IC_{50}) of L435-3 or zanamivir against WSN or PR8 virus infection was examined by plaque assay. All experiments were performed in triplicates. Results were evaluated by GraphPad prism software. [a]SI, selection index, $SI=TC_{50}/IC_{50}$.

Supplementary Figures

Figure S1

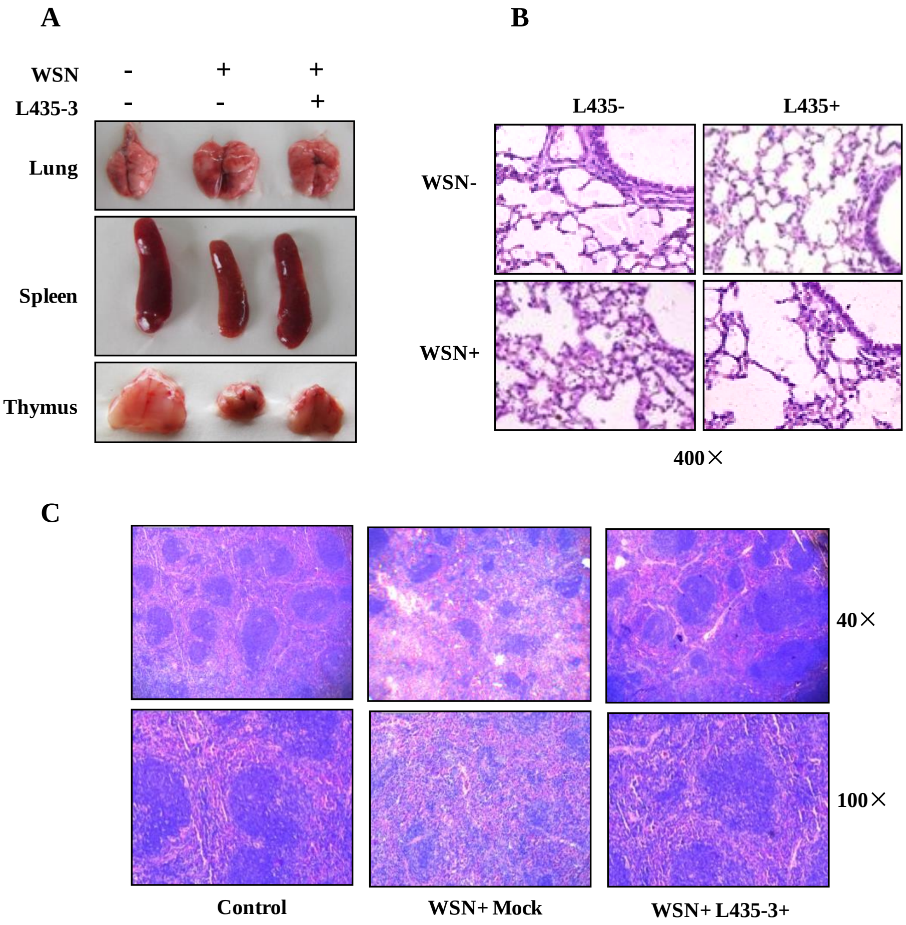

Figure S1. Effect of L435-3 on the pathological changes induced by IAV infection. (A) Control mice and WSN-infected mice were mock treated or treated with L435-3 (0.3 mg/kg/mouse) for 3 days. Then mice were sacrificed, and the lungs, spleens and thymuses were collected. Shown are representative images from three independent experiments. (B, C) Control mice and WSN-infected mice were mock treated or treated with L435-3 as described in (A). Then mice were sacrificed and shown are representative micrographs of mouse lungs (B) and spleens (C) stained with haematoxylin and eosin (H&E).

Figure S2

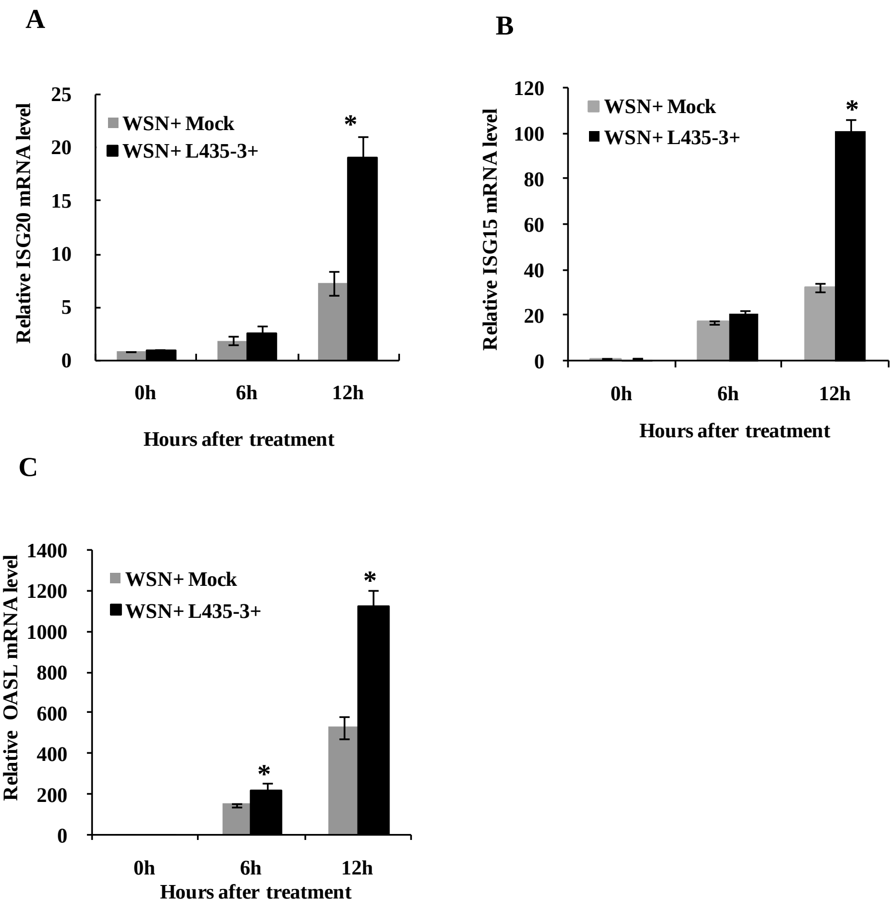

Figure S2. Upregulation of ISG mRNA levels under L435-3 treatment. (A, B and C) WSN-infected A549 cells were treated with or without L435-3 (0.5 μM) for 6 h and 12 h, and then the mRNA levels of *ISG20* (A), *ISG15* (B) and *OASL* (C) were analyzed by quantitative real-time PCR.
